# Acrobatic training prevents learning impairments and astrocyte remodeling in the hippocampus of rats undergoing chronic cerebral hypoperfusion: sex-specific benefits

**DOI:** 10.3389/fresc.2024.1375561

**Published:** 2024-06-13

**Authors:** Ana Paula Rodrigues Martini, Livia Machado Schlemmer, Joelma Alves Lucio Padilha, Rafael Bandeira Fabres, Natividade de Sá Couto Pereira, Lenir Orlandi Pereira, Carla Dalmaz, Carlos Alexandre Netto

**Affiliations:** ^1^ Graduate Program in Neuroscience, Institute of Basic Health Sciences, Universidade Federal do Rio Grande do Sul, Porto Alegre, Brazil; ^2^ Department of Biochemistry, Institute of Basic Health Sciences, Universidade Federal do Rio Grande do Sul, Porto Alegre, Brazil; ^3^ Graduate Program in Biochemistry, Institute of Basic Health Sciences, Universidade Federal do Rio Grande do Sul, Porto Alegre, Brazil; ^4^ Department of Pediatrics, NorthShore University HealthSystem, Evanston, IL, United States; ^5^ Pritzker School of Medicine, University of Chicago, Chicago, IL, United States; ^6^ Psychological Neuroscience Laboratory, Psychology Research Center, School of Psychology, University of Minho, Braga, Portugal

**Keywords:** chronic cerebral hypoperfusion, acrobatic exercise, astrogliosis, hippocampus, sexual dimorphism, cognitive damage

## Abstract

**Background:**

Chronic cerebral hypoperfusion (CCH) leads to memory and learning impairments associated with degeneration and gliosis in the hippocampus. Treatment with physical exercise carries different therapeutic benefits for each sex. We investigated the effects of acrobatic training on astrocyte remodeling in the CA1 and CA3 subfields of the hippocampus and spatial memory impairment in male and female rats at different stages of the two-vessel occlusion (2VO) model.

**Methods:**

Wistar rats were randomly allocated into four groups of males and females: 2VO acrobatic, 2VO sedentary, sham acrobatic, and sham sedentary. The acrobatic training was performed for 4 weeks prior to the 2VO procedure. Brain samples were collected for morphological and biochemical analysis at 3 and 7 days after 2VO. The dorsal hippocampi were removed and prepared for Western blot quantification of Akt, p-Akt, COX IV, cleaved caspase-3, PARP, and GFAP. GFAP immunofluorescence was performed on slices of the hippocampus to count astrocytes and apply the Sholl's circle technique. The Morris water maze was run after 45 days of 2VO.

**Results:**

Acutely, the trained female rats showed increased PARP expression, and the 2VO-trained rats of both sexes presented increased GFAP levels in Western blot. Training, mainly in males, induced an increase in the number of astrocytes in the CA1 subfield. The 2VO rats presented branched astrocytes, while acrobatic training prevented branching. However, the 2VO-induced spatial memory impairment was partially prevented by the acrobatic training.

**Conclusion:**

Acrobatic training restricted the astrocytic remodeling caused by 2VO in the CA1 and CA3 subfields of the hippocampus. The improvement in spatial memory was associated with more organized glial scarring in the trained rats and better cell viability observed in females.

## 1 Introduction

Persistent reduction of blood flow to brain tissue characterizes chronic cerebral hypoperfusion (CCH). It is a condition related to the development and progression of neurodegenerative diseases and cognitive decline in vascular dementia (1), which is the second most common type of dementia (2). CCH is modeled in rodents by the permanent occlusion of the common carotid arteries (2VO, two-vessel occlusion model), culminating in pyramidal neuron loss in the CA1 subfield of the hippocampus and, consequently, memory impairment (3, 4). The cognitive impairment caused by CCH is well documented in the literature and has been reproduced in studies from our laboratory (5–7).

CCH develops in different phases, with the onset phase characterized by acute ischemia persisting for up to 2–3 days (4). Neuronal death begins during ischemia and may extend through the chronic phase (ranging from 8 weeks to 3 months), depending on the extent of compromised cerebral perfusion (6). Reports have shown that the immediate ischemic neuronal death in the CA1 subfield of the hippocampus occurs due to different cellular processes, such as apoptosis and degeneration, and these events are sex-dependent (8). Spatial memory impairment has been shown in the chronic phase, following 2VO, even after the restoration of collateral blood flow (9). Many reports have described neuronal death resulting from CCH in the CA1 subfield (4) due to selective vulnerability (10); however, recent investigations into the CA3 subfield have also been conducted in a CCH model, which despite its lower sensitivity to ischemia (11) plays an important role in the trisynaptic circuit of memory formation (12, 13). Despite the evidence, the complete molecular and cellular mechanisms involved in CA1 and CA3 neuronal death after 2VO remain unclear.

CCH also disturbs astrocyte function in some brain areas, including the CA1 and CA3 subfields of the hippocampus in rodents (14, 15). Reactive astrogliosis has been observed 7 days after 2VO in CA1, suggesting that this phenomenon begins in the acute phase and can last for months (6). Meanwhile, the CA3 subfield showed better adaptive capacity to CCH than CA1, possibly due to changes in astrocytes and microglial cells (15). In reactive astrogliosis, cellular hypertrophy or cellular proliferation may occur, leading to the formation of tissue scars associated with functional impairment (16). Hypertrophic processes of astrocytes overlap around 2VO-affected neurons in the CA1 subfield of the hippocampus in the chronic phase (17). Recently, the modulation of astrocytes by sexually dimorphic characteristics and acrobatic training was demonstrated, showing longer ramifications in male rats compared to female rats after the 2VO model (18). Exercise influences changes in astrocytes, resulting in the improvement of cognitive deficits caused by neurodegenerative and neurovascular diseases (19), promoting the transformation of these activated astrocytes, even in cases of CCH (20).

It is recognized that men and women present differences in susceptibility to dementia and its progression, possibly due to the effects of menopause/andropause (21). Despite neurodegeneration and clinical symptoms of dementia progresses faster in elderly women, men have shorter survival times (22). Physical exercise is a preventive and neuroprotective strategy for several diseases, including dementia, and aerobic exercise is described and widely recommended by clinicians for memory improvement (23). Life expectancy of the population is increasing, and, consequently, it is necessary to anticipate the emergence of chronic age-related diseases (24). Routine practice of exercise seems to be a viable, preventative option for the cognitive impairment caused by dementia (23). Furthermore, evidence points to the association between physical activity and increased perfusion with better cognitive performance in women, as compared to men (25). Therefore, it is understood that physical exercise may have sex-dimorphic effects, which requires further investigation.

Since the prescription of physical exercise, whether for treatment or prevention, appears as an effective tool for neurological diseases and their resulting disabilities, the investigation of molecular changes resulting from exercise could lead to new pharmacological targets (26). A treadmill aerobic protocol applied pre- and post-2VO is effective in preventing spatial memory impairment in rodents (27). Interestingly, acrobatic training is an option that integrates cognitive and motor aspects in different tasks within a circuit, improving coordination and problem-solving performance (28). It was recently shown that acrobatic training is effective in reversing the damage caused by the 2VO when applied in the chronic phase, leading to the recovery of 2VO-induced learning and spatial memory deficits (18).

There is growing interest in the research of cellular and molecular mechanisms involved in cell death and tissue recovery after ischemic insults, especially with regard to sex-related differences (29). Despite existing reports, much remains to be elucidated in this field of research. Therefore, the present study aimed to investigate the effects of preventive acrobatic training on astrocyte remodeling in CA1 and CA3 subfields of the hippocampus and the spatial memory impairment in male and female rats at different stages of the 2VO model. It is hypothesized that there would be differences between the sexes in the parameters evaluated and that acrobatic training will prevent 2VO-induced spatial memory impairment due to astrocyte remodeling in the hippocampus.

## 2 Materials and methods

### 2.1 Ethics and animals

The present study received approval from the Institutional Ethics Committee of the Universidade Federal do Rio Grande do Sul (CEUA/UFRGS protocol #34309), and all procedures followed the National Institutes of Health Guide for the Care and Use of Laboratory Animals, the Federation of Brazilian Societies of Experimental Biology, and the CONCEA guidelines (Brazilian law no. 11,794/2008). All recommendations were followed to minimize the suffering of the animals during the experiments. The surgical procedure was performed by an experienced and trained investigator, and the health condition of the animals was evaluated by the Central Animal House veterinarian. Sedation, analgesia, or anesthesia prescriptions were made whenever necessary.

Two hundred and eight 60-day-old Wistar rats (104 males, approximately 300 g, and 104 females, approximately 200 g) from Central Animal House of the Department of Biochemistry of Universidade Federal do Rio Grande do Sul were used in this experiment. The animals were housed in home cages under standard conditions as follows: 12 h light/dark cycle, humidity and temperature controlled (21 ± 2°C), with water and food *ad libitum*, and the health conditions of the animals frequently evaluated by the Central Animal House veterinarian. All experiments were conducted in the morning (in the light cycle).

### 2.2 Experimental design and groups

**Figure 1:**
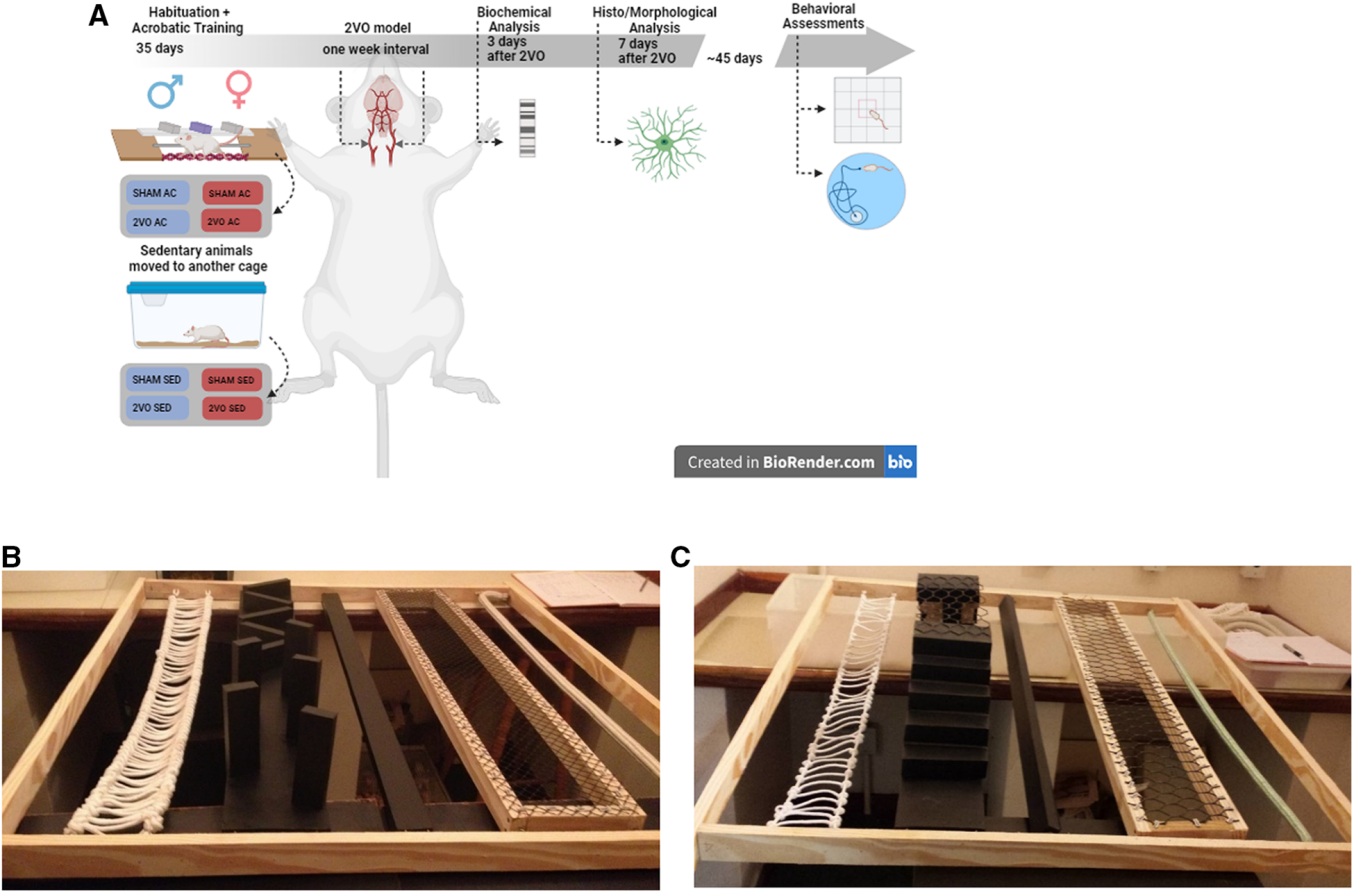
Experimental design (**A**) and acrobatic training circuit (**B,C**). After 4 weeks of acrobatic training on alternate days, 2VO surgery was performed with a 1-week interval between occlusions. The animals were evaluated on the 3rd, 7th, and 45th days after 2VO. Circuit in the first and second weeks (**B**) and circuit in the third and fourth weeks (**C**). The image of the experimental design of this study was created using the free version of BioRender.com available on the Internet.

The animals were randomly assigned to each experimental group by a blinded investigator who generated a list of allocations for every experimental unit (rat) by drawing distinct numbers for the groups studied. Four rats from different experimental groups were randomly allocated to each home cage. The rats were habituated to the acrobatic apparatus for 1 week and then submitted to the acrobatic training protocol for 4 weeks. At the end of the acrobatic training, the rats were submitted to right common carotid artery occlusion or sham surgery. After a 1-week interval, the left common carotid artery was occluded to complete the 2VO procedure or received the sham surgery (described in more detail below). The samples were collected 3 and 7 days after the 2VO procedure for biochemical (*n* = 6 per group) and morphological (*n* = 6 per group) analyses, respectively. The remaining rats were maintained for 45 days (*n* = 9–14 per group) to undergo behavioral tests (Figure 1A).

The rats were randomly allocated into four groups of males and four groups of females as follows: SHAM SED M, male + sham surgery + sedentary (*n* = 11); SHAM AC M, male + sham surgery + acrobatic training (*n* = 11); 2VO SED M, male + 2VO surgery + sedentary (*n* = 12); and 2VO AC M, male + 2VO surgery + acrobatic training (*n* = 12) and SHAM SED F, female + sham surgery + sedentary (*n* = 9); SHAM AC F, female + sham surgery + acrobatic training (*n* = 11); 2VO SED F, female + 2VO surgery + sedentary (*n* = 12); and 2VO AC F, female + 2VO surgery + acrobatic training (*n* = 14).

### 2.3 Acrobatic training protocol

The 60-day-old rats were habituated for a week in the acrobatic circuit or housed in home cages. The groups performed the protocol in the acrobatic circuit, consisting of five tasks, namely, (1) thick or thin rope horizontal bridge, (2) obstacle bridge, (3) narrow or wide parallel bar, (4) grid with large and small holes, and (5) thick or thin single rope, according to the literature (28, 30). The circuit was placed 1.5 m above the floor level, in an appropriate room. Each task was 1 m long and was switched every week to increase the difficulty (Figures 1 B,C). The acrobatic training protocol was performed according to the literature (18, 31). Briefly, the rats completed an acrobatic session, three times per week, on alternate days for a total of 12 training sessions. Each training session consisted of five trials in the circuit, totaling 25 m covered per session. While the indicated groups trained, the animals in sedentary conditions remained in their home cages. The time to complete the circuit was recorded.

### 2.4 Two-vessel occlusion surgery (2VO)

At the end of the acrobatic training, the rats were submitted to 2VO or sham surgery in an appropriate room. The rats were weighed and the anesthesia was induced by inhalation of a mixture of isoflurane (Isoforine, Cristália, São Paulo, Brazil) and 4% oxygen gas and then maintained by inhalation of 2% isoflurane delivered through an inhalation anesthesia device (Brasmed, São Paulo, Brazil) for approximately 7–10 min (average procedure time). The animals were placed in the supine position on a thermal plate (Insight, São Paulo, Brazil) to maintain a temperature of 36°C to avoid hypothermia, with temperature being monitored by a rectal thermometer. Under general anesthesia, an incision was made in the midline of the neck, and the sternocleidomastoid muscle was retracted to locate the right common carotid artery. The Vagus nerve was gently separated, and permanent artery occlusion was performed using a 5–0 silk suture. One week later, the same procedure was performed on the left common carotid artery (5). At the end of the procedure, the animals were sutured and housed in an appropriate room and received postoperative care such as a topical application of 10% lidocaine and oral analgesic medication, in accordance with the instructions of the Central Animal House veterinarian. The mortality rate of the 2VO procedure was approximately 10% (5); therefore, the final number of animals per group may vary. The sham rats received the same procedure, without occlusion of the common carotid arteries.

### 2.5 Biochemical assessments

#### 2.5.1 Western blot

The animals were decapitated 3 days after the 2VO procedure, and samples of both the bilateral and dorsal hippocampi from six rats per group were collected and stored at −80°C. For this analysis, the samples were weighed, and a lysis protocol for protein extraction was applied, which consisted of mechanical maceration in 1:8 NEB buffer (10 mM HEPES, 10 mM KCl, 0.6 mM EDTA at pH = 7.9) to which 1% Nonidet P-40 substitute (Cat# E109, Amresco, USA), 0.25% sodium deoxycholate (Cat# D6750-25G, Sigma-Aldrich, USA), protease inhibitor cocktail (Cat# 11697498001, Roche, Germany), 5 mM sodium fluoride (Cat# 201154-100G, Sigma-Aldrich, USA), and 1 mM sodium orthovanadate were added. Tissue extracts were centrifuged at 960×*g* for 10 min at 4°C, and supernatants were collected. The concentration of total proteins in the supernatant was determined using a commercial bicinchoninic acid (BCA) protein assay kit (Pierce, Thermo Fisher Scientific, USA). After denatured and reduced with lithium dodecyl sulfate (LDS) and 5% mercaptoethanol, the samples were loaded (40 µg protein/lane) on NuPAGE precast 4%–12% gradient polyacrylamide gels (#NP0323BOX, Thermo Fisher Scientific, Waltham, MA, USA), together with a 3.5–260 kDa molecular weight marker (#LC5800, Thermo Fisher Scientific, MA, USA). Electrophoresis and electrotransfer were performed on an XCell SureLock Mini-Cell and XCell IITM Blot Module, respectively (#EI0002, Invitrogen, Thermo Fisher Scientific, Waltham, MA, USA).

Proteins were transferred to nitrocellulose membranes (#10600002, GE HealthCare, Germany) during 1 h 50 at 50 V in transfer buffer (48 mM Trizma, 39 mM glycine, 20% methanol, 0.25% SDS), and membranes were then blocked for 2 h in Tris-buffered saline containing Tween 20 (T-TBS) and 5% (m/v) non-fat dry milk or 5% albumin (32). The membranes were incubated overnight, at 4°C, with the following primary antibodies: anti-cleaved caspase-3 (1:1,000, Cell Signaling Technology Cat# 9661, RRID: AB_2341188), anti-caspase 3 (1:1,000, Cell Signaling Technology Cat# 9661, RRID: AB_2341188), anti-COX IV (subunit of cytochrome c oxidase) (1:1,000, Cell Signaling Technology Cat# 4844, RRID: AB_2085427), anti-PARP poly(ADP-ribose) polymerase (1:1,000, Cell Signaling Technology Cat# 9542, RRID: AB_2160739), anti-p-Akt (phosphorylated Akt) (1:1,000, Cell Signaling Technology Cat# 4060, RRID: AB_2315049), anti-Akt (1:1,000, Cell Signaling Technology Cat# 4685, RRID: AB_2225340), anti-GFAP (glial fibrillary acidic protein) (1:1,000, Thermo Fisher Scientific Cat# MA5-12023, RRID: AB_10984338), and anti-β-actin (1:20,000, Proteintech Cat# HRP-60008, RRID: AB_2819183). β-Actin was used as a loading control. Secondary peroxidase-conjugated anti-rabbit antibody (1:1,000, Sigma-Aldrich Cat# AP132P, RRID: AB_90264) or anti-mouse antibody (1:1,000, Millipore Cat# 402335-2ML, RRID: AB_437955) was incubated for 2 h at room temperature (23°). Chemiluminescence signals were detected using a Pierce ECL Western Kit (#32106; Thermo Fisher Scientific), and images were digitally acquired using the Image Quant LAS 4010 system (GE HealthCare Life Sciences).

### 2.6 Morphological assessments

#### 2.6.1 Immunofluorescence

Six rats per group were deeply anesthetized with inhaled isoflurane (Isoforine, Cristália, São Paulo, Brazil) and euthanized 7 days after 2VO surgery. Posteriorly, 1,000 IU heparin (Cristália, São Paulo, Brazil) was injected via the left ventricle. Subsequently, the rats underwent transcardiac perfusion via the left ventricle with saline solution (0.9% NaCl), followed by 4% paraformaldehyde diluted in 0.1 M phosphate buffer (pH 7.4) at room temperature and pumped using a peristaltic pump (Milan, Paraná, Brazil). The brains were removed from the skulls and samples were post-fixed in the previous fixative solution and cryoprotected with a 15% and 30% sucrose (Neon, Espírito Santo, Brazil) and phosphate buffer saline (PBS) solution. After cryoprotection, the brains were frozen using isopentane, cooled in liquid nitrogen, and stored in a −80°C freezer. The samples were cut in a cryostat (Leica Microsystems, Wetzlar, Germany), and the slices of 30 µm each were sliced and stored in plates containing wells with PBS buffer. The slices were obtained from the dorsal hippocampus region, positioned approximately between the bregma −2.80 mm to −3.60 mm, according to The Rat Brain in Stereotaxic Coordinates (33).

The free-floating immunofluorescence protocol was applied as follows: the samples were heated in 10 mM citrate buffer at 60°C in a water bath for epitope reactivation. One hour later, they were washed in PBS to remove the citrate buffer residue, incubated in a solution containing PBS–Triton X-100 0.3% for 10 min, and incubated in a solution containing 3% goat serum (#A2153, Sigma-Aldrich) diluted in 0.3% PBS–Triton X-100 at room temperature for 45 min (solution to block unspecific binding). Subsequently, double staining was performed on the slices incubated overnight with the primary antibody: GFAP anti-mouse (1:500; Thermo Fisher Scientific Cat# MA5-12023, RRID: AB_10984338). The next day, the slices were washed in PBS and incubated with secondary antibody conjugate with Alexa Fluor 488 anti-mouse (1:800; Thermo Fisher Scientific Cat# A-11001 RRID: AB_2534069) for 1 h in a dark room (room temperature). The slices were washed for the last time in PBS and mounted on gelatin-coated slides covered with coverslips and aqueous mounting medium with DAPI (Fluoroshield F6057, Sigma-Aldrich). The Olympus FV300 confocal microscope equipped with an excitation wavelength of 488 nm was used to visualize the CA1 and CA3 subfields of the hippocampus in 400-fold magnification. All conditions were the same during the capture process.

##### 2.6.1.1 Astrocyte counting

To estimate the number of astrocytes, we performed a semiquantitative count of GFAP-positive cells in the CA1 and CA3 subfields of the hippocampus using an ImageJ cell counter plugin (free download from https://imagej.net/Downloads) (18). Slices were stained by the anti-GFAP immunofluorescence protocol described above using GFAP anti-mouse primary antibody (1:500; Thermo Fisher Scientific Cat# MA5-12023, RRID: AB_10984338) and secondary antibody conjugate with Alexa Fluor 488 anti-mouse (1:800; Thermo Fisher Scientific Cat# A-11001 RRID: AB_2534069). Samples from six animals per group were used to estimate the number of astrocytes in the CA1 and CA3 subfields of the hippocampus. The histological slides contained three slices per rat, and six photomicrographs of each region were captured (36 images/experimental group) by the Olympus FV300 confocal microscope equipped with an excitation wavelength of 488 nm (40× objectives). Only GFAP-positive cells with an immunolabeled body were included in the analysis (34). The mean number of astrocytes per rat was used to estimate the total number of cells in the fields of interest. Data were reported as mean ± SEM.

##### 2.6.1.2 Astrocyte morphology

The assessment of astrocyte morphology was performed on six samples per group in the CA1 and CA3 subfields of the hippocampus. Astrocytes were stained using GFAP anti-mouse primary antibody (1:500; Thermo Fisher Scientific Cat# MA5-12023, RRID: AB_10984338) and secondary antibody conjugate with Alexa Fluor 488 anti-mouse (1:800; Thermo Fisher Scientific Cat# A-11001 RRID: AB_2534069). Photomicrographs were captured by the Olympus FV300 confocal microscope equipped with an excitation wavelength of 488 nm (40× objectives). Sholl's concentric circles method was performed according to the literature (35): eight concentric circles were drawn with 6 µm intervals around each cell analyzed. Three astrocytes were analyzed per image, evaluating the number of intersections of the primary astrocytic process and the length of the astrocytic processes in both hemispheres. We standardized our analysis to eight cycles, thus limiting the analyzed perimeter to 48 μm per cell. Analyzes were quantified using the Image-Pro Plus 6.0 software (18, 35–37). Data were reported as mean ± SEM.

### 2.7 Behavioral assessments

#### 2.7.1 Open-field task

At the end of 45 days, the rats' spontaneous ambulation activity was evaluated in the open-field test. Black wooden boxes measuring 60 cm  ×  45 cm × 30 cm were used in the test. Room conditions such as lighting, temperature, and noise were controlled. Each test lasted 5 min and was filmed by a camera positioned on the roof connected to the computer with the ANY-maze software. The following parameters were evaluated: total distance traveled, number of crossings, time spent in the central zone, and time spent in the peripheral zone by each animal. Data were reported as mean ± SEM (5).

#### 2.7.2 Morris water maze task

The Morris water maze was used to assess the different aspects of memory and learning. The apparatus consisted of a circular pool, measuring 200 cm in diameter × 100 cm in height × 40 cm deep, filled with water at approximately 23°C. The pool was virtually divided into north, south, east, and west. Inside the pool, a platform submerged 2 cm below the water surface was placed. Visual cues were placed on the walls of the room to guide the animals to the platform during memory acquisition. Each training session was filmed by a camera on the roof overlooking the pool, connected to the computer, images were acquired using the ANY-maze software.

##### 2.7.2.1 Reference memory protocol (acquisition)

After the open-field task, the rats proceeded to the water maze test. The animals were submitted to the reference memory protocol, in which they were trained for six consecutive days. Each day, the animals were placed in the pool facing the wall in four different initial positions (north, south, east, and west of the tank), and 60 s was stipulated for the rats to find the submerged platform (which was always in the same position during the training period). When the animals did not find the platform, they were gently guided to the platform where they remained for 20 s to learn. The rats performed four trials per day with a 15-min interval between trials. The time spent during training to find the platform was considered an indicator of learning (i.e., the lower the latency to find the platform, the better the learning) (38, 39). The area under the learning curve (AUC) was calculated. The average value of the four daily training sessions was taken for statistical analysis. Data were reported as mean in seconds ± SEM.

##### 2.7.2.2 Probe (memory)

The probe was performed 24 h after the last day of training and evaluated the rats' memory in a single attempt to find the platform within 60 s. The probe session is performed without the platform to assess aspects of the animals' spatial memory. All rats were placed in the pool in the same position equidistant from the place where the platform would be. The parameters evaluated were time spent in the target quadrant, time spent in the platform zone, total distance traveled, and latency to find the platform. Data were reported as mean ± SEM.

### 2.8 Statistical analysis

Sample sizes for behavioral (*n* = 9–14), biochemical, and histological analysis (*n* = 6) were defined based on previous studies from our laboratory (5, 40). The following parameters were used: normal two-tailed distribution, *α* of 0.05%, and 80% power calculated by G*Power. All analyses were run using SPSS 21.0 for the Windows software (SPSS Inc., Chicago, IL, USA), with significance established as *p *≤ 0.05. To verify the normality of the data, we performed a Shapiro–Wilk test, evaluated parametric data by three-way analysis of variance (ANOVA), and applied repeated measures when necessary. Mauchly's test was used to assess data sphericity when using repeated measures ANOVA. If the sphericity assumption was violated (*p* < 0.05), the Greenhouse–Geisser correction was used. The generalized linear model (GzLM) was used followed by the Bonferroni *post hoc* test for non-parametric data. Correlation analysis was performed using the Spearman correlation test. The factors considered were *sex*, *lesion*, and *treatment* and their interactions. Data were reported as mean ± SEM. The graphical representation was obtained by the GraphPad Prism 6 software.

## 3 Results

### 3.1 Baseline data

A total of 208 rats were used in this experiment (104 males and 104 females). Mortality rates after 2VO surgery were 9.61% for males (10 rats) and 9.61% for females (10 rats). After the first occlusion, the males were heavier (381.1 ± 4.4 g) than the females (239.8 ± 4.5 g) (*sex* effect; *p* ≤ 0.05). After the second occlusion, the males remained heavier (386.11 ± 5.2 g) than the females (254.1 ± 5.3 g) (*sex* effect; *p* ≤ 0.05) and also in euthanasia [males (481.4 ± 4.3 g) vs. females (275.4 ± 4.4 g) (*sex* effect; *p* ≤ 0.05)]. As expected, the males were heavier than the females at all phases, and no differences in weight gain were found between both sexes during surgery recovery (*p* < 0.05).

### 3.2 Acrobatic training performance

There were no differences between the two groups in terms of the average time to complete the circuit in the first week; however, in the second, third, and fourth weeks, the females were faster than the males (*sex* effect; *p* ≤ 0.05).

### 3.3 Western blot analysis

**Figure 2:**
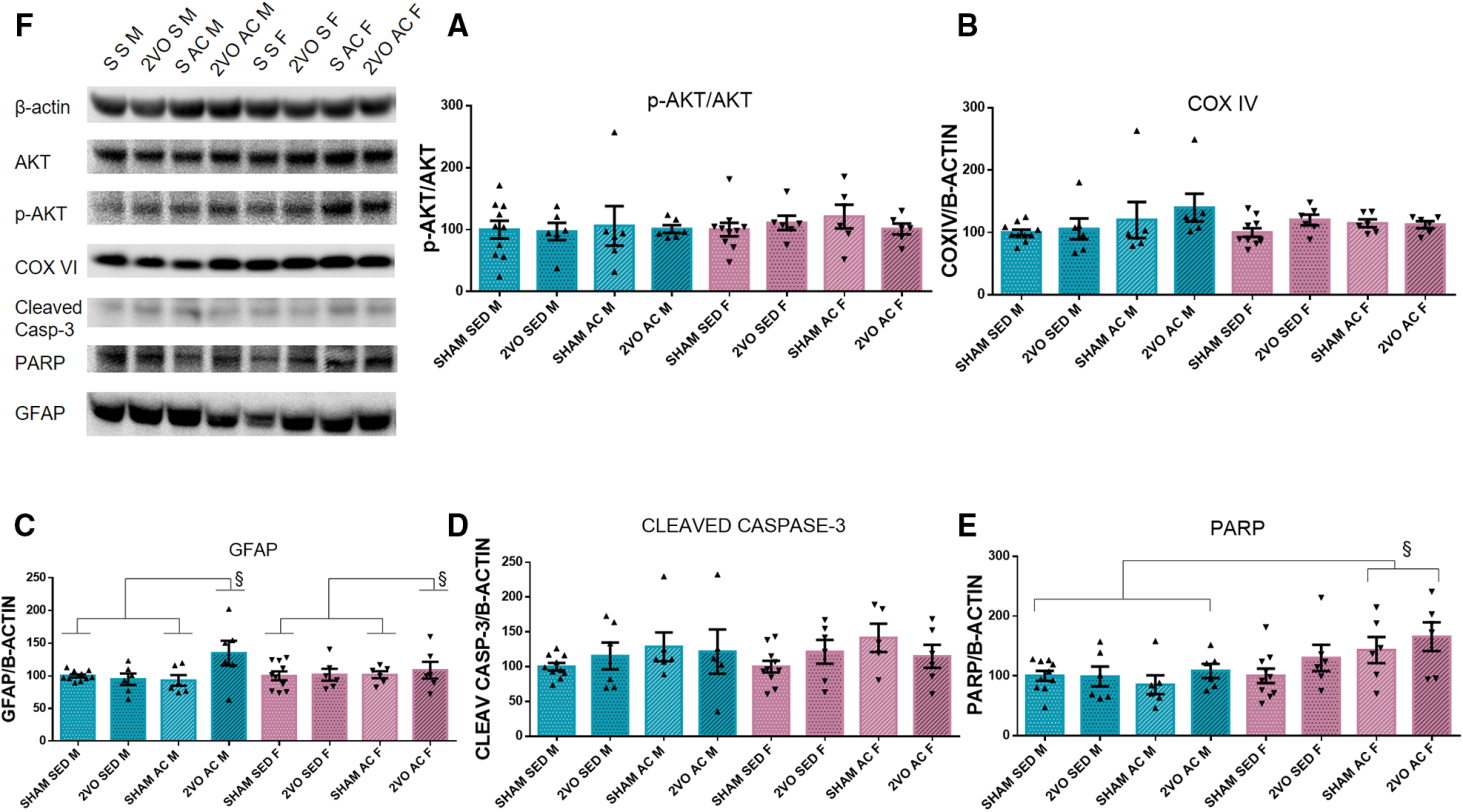
Protein quantification by Western blot 3 days after 2VO and representative images of protein bands (**A**). The ratio of Akt phosphorylated by Akt (**B**), COX IV (**C**), and GFAP (**D**). ^§^The 2VO-trained animals are different from the sham-trained and sedentary sham animals (*sex–treatment* interaction). Cleaved caspase-3 (**E**). PARP (**F**). ^§^The trained females are different from trained males and sedentary males (*sex–treatment* interaction). Blue bars: male groups. Purple bars: female groups. Statistics by GzLM followed by Bonferroni's *post hoc*. *p* ≤ 0.05; *n* = 6 per group. The results are expressed as mean ± SEM. The group labels are described in the Materials and methods section.

On the third day after 2VO, there were no differences for COX IV, cleaved caspase-3, and p-Akt/Akt ratio. The acrobatic females (154.30 ± 11.27) expressed more PARP compared to the acrobatic (96.72 ± 11.27) and sedentary males (99.48 ± 10.08) (*sex–treatment* interaction; *p* ≤ 0.05). The acrobatic 2VO animals showed increased GFAP expression (121.78 ± 6.44) compared to the acrobatic sham (97.30 ± 6.44) and sedentary sham animals (100 ± 4.99) (*lesion–treatment* interaction; *p* ≤ 0.05) (Figures 2A–F). The GFAP increase in the acute phase of 2VO was induced by acrobatic training, and the PARP increase in females suggests a sex-specific effect of cell protection.

### 3.4 Immunofluorescence in the CA1 and CA3 subfields of the hippocampus

#### 3.4.1 Astrocyte counting

**Figure 3:**
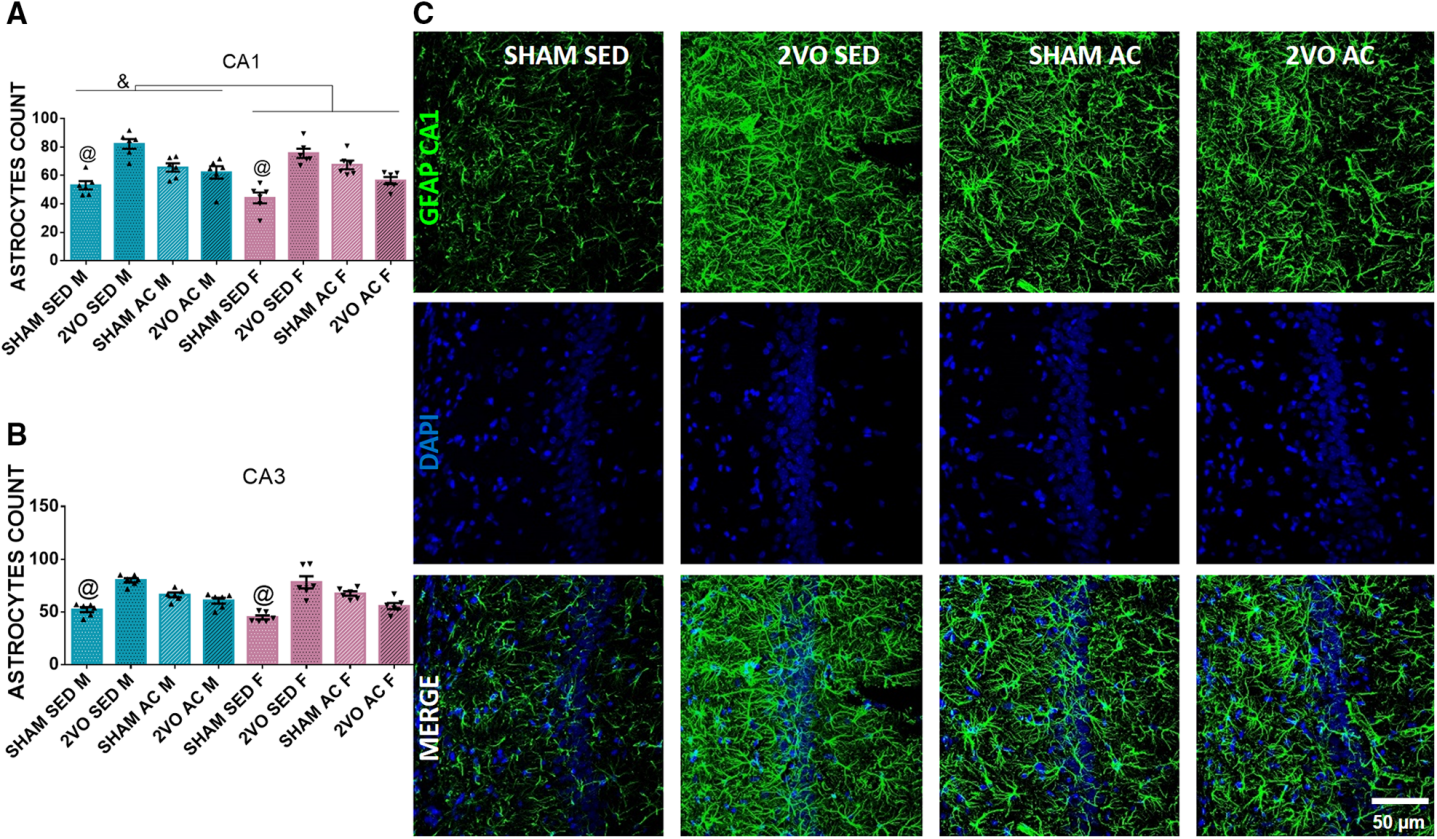
Morphological analysis of astrocytes 7 days after 2VO. Astrocytes count in the CA1 (**A**) and CA3 subfields (**B**). ^&^Males vs. females (sex effect). ^@^The sedentary sham animals are different compared to others (*lesion–treatment interaction*). Representative image of astrocytes 7 days after 2VO in the CA1 subfield (**C**). Blue bars: male groups. Purple bars: female groups. Statistics by GzLM followed by Bonferroni's *post hoc*. *p* ≤ 0.05; *n* = 6 per group. The results are expressed as mean ± SEM. The scale bar is equivalent to 400 µm. The group labels are described in the Materials and methods section.

On the seventh day after 2VO, the males had more astrocytes (65.73 ± 1.52) than the females (60.90 ± 1.52) in the CA1 subfield 7 days after 2VO (*sex* effect; *p* ≤ 0.05). In addition, the sedentary sham animals (48.62 ± 2.15) had fewer astrocytes than the other groups (*lesion–treatment* interaction; *p* ≤ 0.05). In addition, in the CA3 subfield, the sedentary sham animals had fewer astrocytes (48.55 ± 1.93) than the other groups (*lesion–treatment* interaction; *p* ≤ 0.05) (Figures 3A–C). The increase in the number of astrocytes appeared to be a consequence of both 2VO insult and acrobatic training, mainly in the CA1 subfield for males.

#### 3.4.2 Sholl's circle (astrocyte analysis)

**Figure 4:**
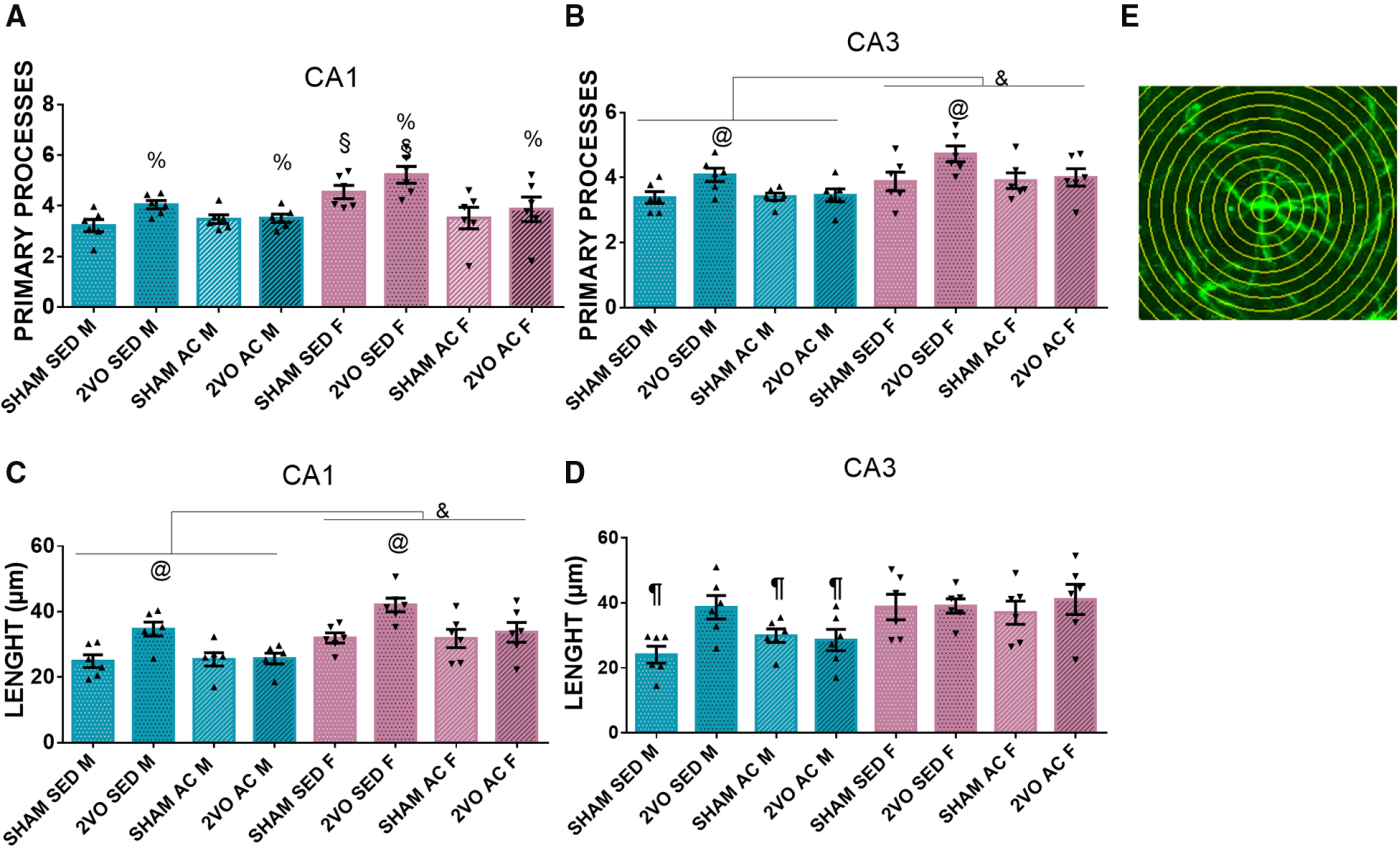
Number of astrocyte primary processes in the CA1 (**A**) and CA3 subfields (**B**). ^&^Males vs. females (sex effect). ^@^The sedentary 2VO animals are different compared to others (*lesion–treatment interaction*). ^%^2VO animals vs. sham (lesion effect). ^§^The sedentary females are different compared to others (*sex–treatment interaction*). Length of astrocytic processes in the CA1 (**C**) and CA3 (**D**) subfields. ^&^Males vs. females (sex effect). ^@^The sedentary 2VO animals are different compared to others (*lesion–treatment interaction*). ^¶^The sedentary sham male group is different from others, except the acrobatic sham male and acrobatic 2VO male groups (*lesion–sex–treatment interaction*). Representative image of Sholl's concentric circles method (**E**). Blue bars, male groups. Purple bars, female groups. Statistics by GzLM followed by Bonferroni's *post hoc*. *p* ≤ 0.05; *n* = 6 per group. The results are expressed as mean ± SEM. The scale bar is equivalent to 400 µm. The group labels are described in the Materials and methods section.

On the seventh day after 2VO, the 2VO animals had more primary processes (4.16 ± 0.13) than the sham animals (3.69 ± 0.13) (*lesion* effect; *p* ≤ 0.05). In addition, the sedentary females presented more primary processes (4.88 ± 0.19) than the other groups (*sex–treatment* interaction; *p* ≤ 0.05). In the CA3 subfield, the females had more primary processes (4.12 ± 0.10) than the males (3.58 ± 0.10) (*sex* effect; *p* ≤ 0.05). Furthermore, the sedentary 2VO animals (4.40 ± 0.14) had more primary processes than the other groups (*lesion–treatment* interaction; *p* ≤ 0.05) (Figures 4A,B).

In the CA1 subfield, the females had longer astrocyte processes (34.89 ± 1) than the males (27.67 ± 1) (*sex* effect; (*p* ≤ 0.05), and the sedentary 2VO animals had longer processes (38.37 ± 1.42) than the other groups (*lesion–treatment* interaction; *p* ≤ 0.05). In the CA3 subfield, the sedentary sham male group (24.08 ± 3.04) had shorter primary processes than the other groups, except the acrobatic sham male group (29.94 ± 3.04) and acrobatic 2VO male group (28.58 ± 3.04) (*lesion–sex–treatment* interaction; *p* ≤ 0.05). The 2VO animals presented with longer branches in astrocytes, and the acrobatic training prevented the branching in males and females in CA1 and partially in CA3. Additionally, sedentary females presented with more primary processes (Figures 4C–E).

### 3.5 Open field

In the open-field task, the females (15.89 ± 0.51 m) traveled greater distances compared to the males (10.76 ± 0.51 m) (*sex* effect; *p* ≤ 0.05). Moreover, the trained animals (12.48 ± 0.50 m) traveled less than the sedentary ones (14.17 ± 0.52 m) (*treatment* effect; *p* ≤ 0.05). The females (135.79 ± 5.07) crossed more than the males (106.44 ± 5) (*sex* effect; *p* ≤ 0.05), and the trained animals (113.35 ± 4.93) crossed fewer than the sedentary ones (128.87 ± 5.16) (*treatment* effect; *p* ≤ 0.05) (Table 1).

**Table 1:** The results of the open-field test and probe test performed in the Morris water maze.

Males	SHAM SED (*N* = 11)	2VO SED (*N* = 12)	SHAM AC (*N* = 11)	2VO AC (*N* = 12)
Open field				
Distance traveled (m)	12.66 ± 1	10.48 ± 1	10.17 ± 1^b^	9.72 ± 1^b^
Crossings	118.81 ± 10.25	108 ± 9.8	108.36 ± 10.25^b^	90.58 ± 9.82^b^
Time in the peripheral zone (s)	277.04 ± 3.31	282.60 ± 3.17	276.61 ± 3.31	276.32 ± 3.17
Time in the central zone (s)	22.95 ± 3.31	17.39 ± 3.17	23.38 ± 3.31	23.67 ± 3.17
Water maze				
Time in the target quadrant (s)	27.69 ± 2.8	22.60 ± 2.6	21.83 ± 2.8	25.29 ± 2.7
Time in the platform zone (s)	0.7 ± 0.14^a^	0.4 ± 0.14^a^	0.5 ± 0.14^a^	0.6 ± 0.14^a^
Latency to find the platform (s)	35.51 ± 5.7	46.25 ± 5.4^c^	33.94 ± 5.7	38.76 ± 5.4^c^
Total distance traveled (m)	3.9 ± 0.3	3.7 ± 0.3	4.2 ± 0.3	4.7 ± 0.3
Swimming speed (m/s)	0.06 ± 0.006	0.06 ± 0.006	0.07 ± 0.006	0.07 ± 0.006
Females	SHAM SED (*N* = 9)	2VO SED (*N* = 12)	SHAM AC (*N* = 11)	2VO AC (*N* = 14)
Open field				
Distance traveled (m)	15.44 ± 1.1^a^	18.1 ± 1^a^	15.38 ± 1^a^^,^^b^	14.65 ± 0.92^a^^,^^b^
Crossings	146.77 ± 11.33^a^	141.91 ± 9.82^a^	112.54 ± 10.25^a^^,^^b^	141.92 ± 9.09^a^^,^^b^
Time in the peripheral zone (s)	279.52 ± 3.66	284.4 ± 3.17	285.58 ± 3.31	279.67 ± 2.93
Time in the central zone (s)	20.47 ± 3.66	15.59 ± 3.17	14.41 ± 3.31	20.32 ± 2.93
Water maze				
Time in the target quadrant (s)	25.34 ± 3.1	18.20 ± 2.7	23.56 ± 2.8	18.55 ± 2.5
Time in the platform zone (s)	0.4 ± 0.16	0.1 ± 0.14	0.4 ± 0.14	0.2 ± 0.13
Latency to find platform (s)	37.08 ± 6.3	49.11 ± 5.4^c^	32.9 ± 5.7	46.27 ± 5^c^
Total distance traveled (m)	4.4 ± 0.4^a^	5.1 ± 0.3^a^	5 ± 0.3^a^	4.3 ± 0.3^a^
Swimming speed (m/s)	0.07 ± 0.006^a^	0.08 ± 0.006^a^	0.08 ± 0.006^a^	0.08 ± 0.006^a^

^a^ The sex effect among the female and male groups.

^b^ The treatment effect showing that the acrobatic groups were different compared to the sedentary groups.

^c^ The lesion effect showing that the 2VO groups were different compared to the sham groups. Statistical analysis was performed by generalized linear model followed by Bonferroni's *post hoc*. All values: *p* ≤ 0.05 were assumed*.* Groups: SHAM SED, sham surgery + sedentary treatment; 2VO SED, two-vessel occlusion surgery + sedentary treatment; SHAM AC, sham surgery + acrobatic training; 2VO AC, two-vessel occlusion surgery + acrobatic training. The values are expressed as mean ± SEM.

### 3.6 Morris water maze

#### 3.6.1 Learning (reference protocol)

Repeated measures ANOVA revealed that the animals spent less time finding the platform over the 6 days of training during the learning acquisition phase (*p* ≤ 0.05). The sham animals learned better than the 2VO animals on Days 2, 3, 5, and 6 (*lesion–day* interaction; *p* ≤ 0.05). Additionally, the trained animals learned better than the sedentary animals, and the 2VO animals had worse performance compared to the sham animals (*lesion* and *treatment* effects; *p* ≤ 0.05).

**Figure 5:**
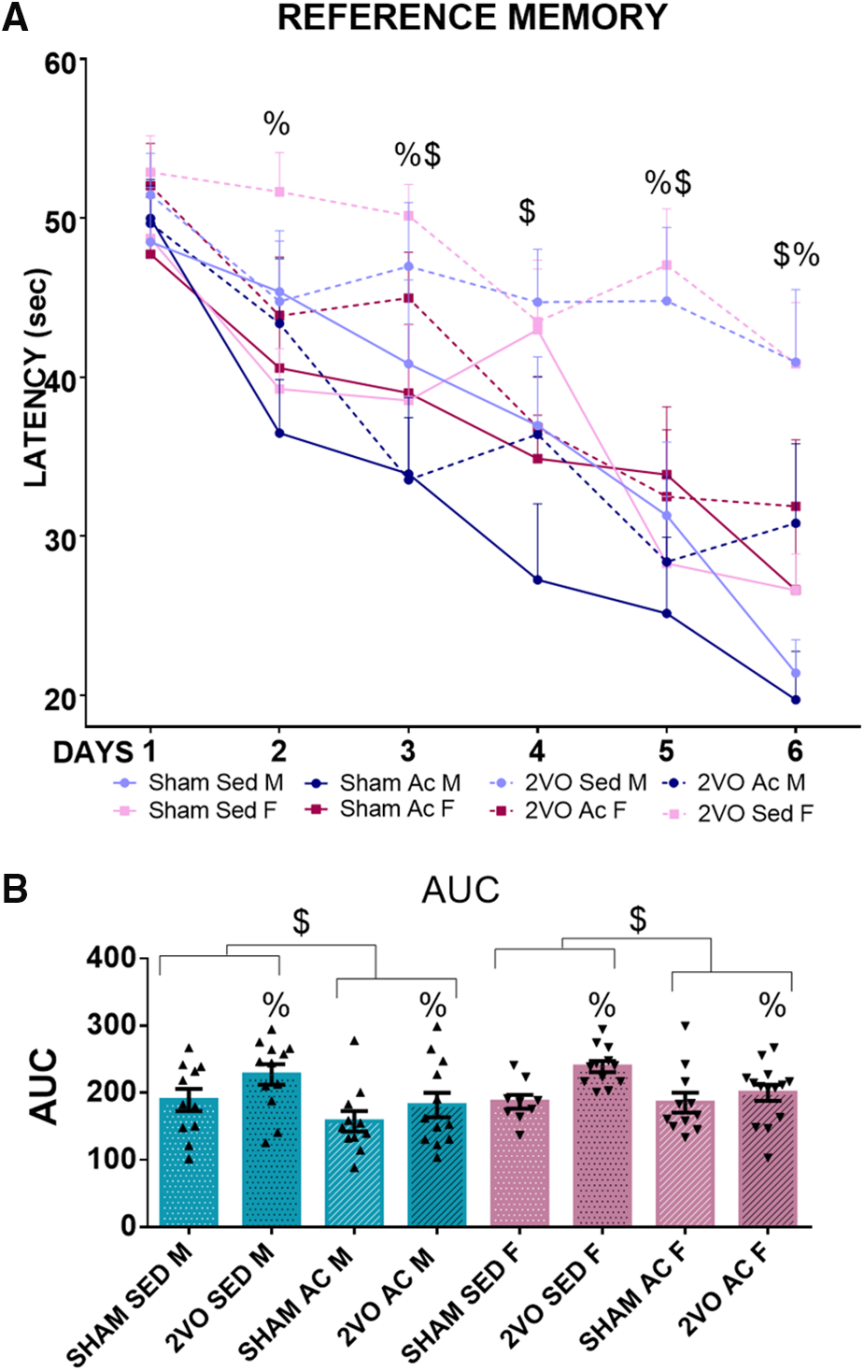
Morris water maze performance during 6 days of training (**A**). ^$^Trained vs. sedentary animals (treatment effect)/ ^&^Males vs. females (sex effect). ^%^2VO animals vs. sham (lesion effect). Blue lines, male groups. Purple lines, female groups. Dotted lines, 2VO groups. Solid lines, sham groups. Repeated measures ANOVA followed by Bonferroni’s *post hoc*. *p* ≤ 0.05. area under the learning curve (AUC) (**B**). Blue bars, male groups. Purple bars, female groups. Statistics by GzLM followed by Bonferroni's *post hoc*. *p* ≤ 0.05; *n* = 9–14 per group. The results are expressed as mean ± SEM. The group labels are described in the Materials and methods section.

In support of these results, the analysis of the AUC showed that the 2VO animals (212.14 ± 6.5 s) had more difficulty in learning than the sham animals (179.78 ± 7.1 s) (*lesion* effect; *p* ≤ 0.05) and the trained animals (181.27 ± 6.7 s) learned faster than the sedentary animals (210.66 ± 7 s) (*treatment* effect; *p* ≤ 0.05) (Figures 5A,B). These results indicate that acrobatic training improved spatial learning and has the potential to prevent the impairment caused by 2VO.

#### 3.6.2 Memory (probe trial)

There was no significant difference in the time spent in the target quadrant (where the platform would be located). However, the 2VO animals (45.10 ± 2.6 s) needed more time to find the platform than the sham animals (34.8 ± 2.9 s) (*lesion* effect; *p* ≤ 0.05). In addition, the males (0.5 ± 0.07 s) spent more time within the platform zone compared to the females (0.3 ± 0.07 s) (*Sex* effect; *p* ≤ 0.05). The total distance swimming in the test showed that the females swam (4.7 ± 0.19 m) more than the males (4.1 ± 0.19 m) (*sex* effect; *p* ≤ 0.05). Similarly, the females swam faster (0.07 ± 0.003 m/s) compared to the males (0.06 ± 0.003 m/s) (*sex* effect; *p* ≤ 0.05). There was no lesion effect on total distance and swimming speed, indicating the absence of motor impairment to perform the task. These results confirm that spatial memory impairment is caused by the 2VO lesion, but unlike what was observed during the learning phase, acrobatic training had limited (if any) benefits in preventing memory damage (Table 1).

### 3.7 Behavioral–morphologic correlations

Correlations were performed between behavioral parameters in the water maze and morphological data. There was a positive correlation between the latency to find the platform on the sixth day of learning and the number of astrocytes and the number of primary processes in the CA1 subfield and length (*p* ≤ 0.05; Table 2), indicating that the animals with longer latencies (spatial learning impairment) showed increases in the number of GFAP-positive cells and branching. Moreover, the greater AUC and the time to reach the platform on probe trial (spatial memory impairment) were correlated with greater numbers of astrocytic branches in CA1 (*p* ≤ 0.05; Table 2).

**Table 2:** Correlation between the learning and memory parameters of the water maze and morphological data.

	Morphological data					
	Primary processes CA1	Primary processes CA3	Length CA1	Length CA3	Astrocyte count CA1	Astrocyte count CA3
Water maze data	Rho	Rho	Rho	Rho	Rho	Rho
Six days of learning	0.427	–	0.347	–	0.275	–
AUC	0.332	–	–	–	–	–
Latency to find the platform	0.368	–	–	–	–	–

The sixth day of learning showed a directly proportional result to the number and length of the astrocytic process and astrocyte count in CA1. Statistics by Spearman correlation. All correlations were considered moderate. All values: *p* ≤ 0.05 were assumed.

## 4 Discussion

The present work provides further evidence of the preventive effects of acrobatic training in attenuating the cognitive damage of CCH by diminishing cell death in the hippocampus and improving late behavioral outcomes, in both male and female rats. In support of the working hypothesis, it is reported that (a) acutely, 3 days after 2VO, an increase in GFAP expression was observed in the trained animals, indicating an astrocytic reaction. Furthermore, the trained females showed a positive effect on cell viability, as suggested by increased PARP expression. (b) Seven days after 2VO, there was an increased number of astrocytes, suggestive of astrogliosis in CA1 and CA3 after 2VO and acrobatic training, mainly for males. However, the 2VO animals showed longer and more branched astrocytes, and acrobatic training prevented branching in the CA1 subfield of females. (c) Finally, 45 days after 2VO, spatial memory was impaired, and acrobatic training partially prevented such deficits.

### 4.1 Three days after 2VO: effects of 2VO and acrobatic training on astrocyte reaction and cell viability in the dorsal hippocampus

Here, a sex-specific effect for the trained females in cell viability was demonstrated, suggested by an increased PARP expression in the hippocampus. PARP repairs DNA damage by adding poly(ADP-ribose) polymers in response to cellular stresses (41). The full-length PARP is characteristic of non-apoptotic cells (42) and also participates in the regulation of astrocyte activation (43) and long-term memory (44, 45). The increased PARP was previously observed 7 days after 2VO in males and females, which was accompanied by a decrease in neurodegeneration in females (8). The increased PARP in the acrobatic females can be attributed in part to the hormonal response, considering the increases in full-length PARP in the mitochondrial fraction detected in male rats treated with 17β-estradiol, suggesting the protection of mitochondrial DNA by inhibiting the cascade involved in the mitochondrial apoptotic pathway in the prefrontal cortex following 2VO (46).

Interestingly, the trained animals showed an increase in the GFAP expression 3 days after 2VO, which could be explained by acrobatic training modulation and the ischemia in parallel. Reactive astrogliosis is reported in the pathogenesis of several neurodegenerative diseases (16) occurring immediately in response to a CCH event (47). Astrocytes react to stimuli through changes in their morphology and function, presenting plasticity (48). Physical exercise influences astrocytes in several aspects (49) and promotes an increased number of new astrocytes, increased glutamate uptake, and release of trophic factors (50). Thus, the increased expression of GFAP in the trained rats can be explained, in part, by the plasticity promoted in response to acrobatic training.

The Akt pathway plays a critical role in controlling neural cell survival and apoptosis (51), due to phosphorylation and inactivation of several pro-apoptotic proteins (52). CCH results in mitochondrial dysfunction and decreased cytochrome oxidase activity leading to deficits in learning and memory (53, 54). Although neuronal death occurs immediately after an ischemic insult, no differences were observed in the p-Akt/Akt ratio, COX function, and cleaved caspase-3 3 days after 2VO in this study.

### 4.2 Seven days after 2VO: effects of 2VO and acrobatic training on astrocyte branching and astrogliosis in the CA1 and CA3 subfields

Pathological conditions trigger morphological, molecular, and functional changes in astrocytes that become reactive (55). According to reports, after 2VO, the rats presented reactive astrocytes with neurotoxic characteristics, but early physical exercise prevents this effect (20, 56). In the present study, the number of astrocytes increased in the CA1 and CA3 subfields caused by 2VO, as assessed 7 days after the event, supporting the hypothesis that 2VO leads to neurotoxic reactive gliosis due to the ischemic insult. The trained animals also showed an increased number of astrocytes associated with cognitive improvement in behavioral tasks, indicating exercise-induced neuroplastic effect in the CA1 and CA3 subfields, as previously observed after ischemia in other hippocampal areas (57) and after 2VO in other brain structures (20, 58). Exercise-induced changes in astrocytes may be a key mechanism for improving cognitive and executive functions (19, 50), playing an important role in neuroplasticity and synaptogenesis and aiding neurorehabilitation (59).

Here, the 2VO animals showed longer and more branched astrocytes, observed mainly in sedentary females, demonstrating a sex-specific effect in the CA1 and CA3 subfields. A recent study in our laboratory correlated the branching in the chronic phase after 2VO with cognitive deficits in the water maze task, indicating a neurotoxic effect in the hippocampus (18). This branching pattern was observed in the rats of both sexes after neonatal hypoxia–ischemia (37) and after experimental intracerebral hemorrhage (35). This set of evidence is suggestive of neurotoxic astrogliosis, possibly modulated by oxidative and inflammatory agents. Disruption of primary astrocyte cilia can lead to gliosis and neuroinflammation (60), increasing the number of C3 (a marker for neurotoxic astrocytes)/GFAP-positive astrocytes in rats 2 months after 2VO (56). In the present study, the 2VO-trained animals also showed branched astrocytes in response to ischemia. However, acrobatic training may have positively modulated the astrocyte reaction, explaining the restoration of learning in the cognitive task. The astrocyte modifications caused by exercise can benefit several aspects of synapses, playing a critical role in memory formation (49). In addition to astrocytes, exercise also influences microglia, promoting neuroprotection in rats after 2VO (20, 61).

### 4.3 The chronic phase: effects of 2VO and acrobatic training on spatial memory

The 2VO rats showed learning impairment in the Morris water maze test, which was prevented by acrobatic training. There was no difference between the control and 2VO groups in terms of the distance traveled in the open-field test and swimming speed in the water maze, indicating that the water maze task results were not biased by a possible motor dysfunction or relevant locomotor difficulty caused by the 2VO model. Spatial learning impairment was expected, as previously reported in the literature (6, 7, 18), probably due to apoptosis and reactive astrocytosis in the CA1 subfield of the hippocampus (4, 14). Here, the sedentary females had longer astrocyte branches than the sedentary males in the acute phase, which has previously been correlated with impairment in the spatial task (18), while acrobatic training prevented such branching patterns in the CA1 subfield of the trained animals.

In the present study, the correlations between the water maze parameters and the morphological data evidenced that the animals with longer latencies showed increased numbers of astrocytes and astrocyte branching in the CA1 subfield, possibly forming a disorganized tissue scar after 2VO, as previously described (17), implying memory impairments (18). It has already been reported that a cognitive rehabilitation protocol including voluntary exercise on a running wheel can attenuate spatial memory impairments and prevent alterations in the CA1 subfield of the hippocampus in 2VO males, but not in 2VO females (62, 63). Despite learning increases in the 6 days of water maze training, in the probe trial, there were no strong signs of memory retrieval. Thus, the acrobatic training partially prevented the spatial memory deficits caused by 2VO, being particularly relevant in the learning phase. A limitation of the present study is the lack of evaluation of the estrous cycle of female rats along with the experiment to better support considerations about the morphological and behavioral changes found. This variable will be pursued in further studies.

### 4.4 Perspectives and future directions

Evidence indicates that physical activity brings benefits and is a widely adopted treatment among patients with dementia (64). Combining physical exercise or an enriched environment with regenerative therapies, such as cell therapy, can mitigate cognitive impairment caused by neurodegenerative diseases by creating a microenvironment that allows transplanted immature cells to learn neurochemical and physiological signals optimizing functional connectivity in the graft-host (65). According to the literature, the use of cell therapy in the treatment of experimental Alzheimer's disease, the most prevalent type of dementia, can promote neurogenesis and synaptogenesis, improving cognition (66–68). Considering such benefits, the association of acrobatic exercise with regenerative cell therapy may facilitate the process of functional integration, connectivity, and adequate differentiation in the host environment, indicating a field of research of potential therapeutic value. According to the emerging literature, we suggest that the effects of acrobatic exercise in preventing cognitive damage will be enhanced by cellular regenerative therapy.

## 5 Conclusion

Early acrobatic training promoted greater cell viability in females in the acute phase of 2VO, possibly contributing to the prevention of neuronal death in the hippocampus and influencing spatial memory. Hypoperfusion caused neurotoxic reactivity of astrocytes represented by increased branching in the CA1 and CA3 subfields of the hippocampus. Exercise induced a neuroplastic effect, which was associated with better performances in learning tasks. Although there were no benefits on memory retrieval during the probe trial correlated to astrocytic scarring, the acrobatic training was effective in the learning phase, through mechanisms that may be linked to astrocyte neuroplasticity and neuronal survival in the CA1 subfield.

## Data Availability

The original contributions presented in the study are included in the article/Supplementary Material, further inquiries can be directed to the corresponding author.
